# MHC Adaptive Divergence between Closely Related and Sympatric African Cichlids

**DOI:** 10.1371/journal.pone.0000734

**Published:** 2007-08-15

**Authors:** Jonatan Blais, Ciro Rico, Cock van Oosterhout, Joanne Cable, George F. Turner, Louis Bernatchez

**Affiliations:** 1 Département de Biologie, Québec-Océan, Université Laval, Quebec, Québec, Canada; 2 Estación Biológica de Doñana, Consejo Superior de Investigaciones Científicas, Sevilla, Spain; 3 Department of Biological Sciences, University of Hull, Hull, United Kingdom; 4 School of Biological Sciences, Cardiff University, Cardiff, United Kingdom; Wellcome Trust Centre for Human Genetics, United Kingdom

## Abstract

**Background:**

The haplochromine cichlid species assemblages of Lake Malawi and Victoria represent some of the most important study systems in evolutionary biology. Identifying adaptive divergence between closely-related species can provide important insights into the processes that may have contributed to these spectacular radiations. Here, we studied a pair of sympatric Lake Malawi species, *Pseudotropheus fainzilberi* and *P. emmiltos*, whose reproductive isolation depends on olfactory communication. We tested the hypothesis that these species have undergone divergent selection at MHC class II genes, which are known to contribute to olfactory-based mate choice in other taxa.

**Methodology/Principal Findings:**

Divergent selection on functional alleles was inferred from the higher genetic divergence at putative antigen binding sites (ABS) amino acid sequences than at putatively neutrally evolving sites at intron 1, exon 2 synonymous sequences and exon 2 amino acid residues outside the putative ABS. In addition, sympatric populations of these fish species differed significantly in communities of eukaryotic parasites.

**Conclusions/Significance:**

We propose that local host-parasite coevolutionary dynamics may have driven adaptive divergence in MHC alleles, influencing odor-mediated mate choice and leading to reproductive isolation. These results provide the first evidence for a novel mechanism of adaptive speciation and the first evidence of adaptive divergence at the MHC in closely related African cichlid fishes.

## Introduction

Explaining why certain ecosystems harbor disproportionately high species richness is a hotly debated issue [1]. Antagonistic coevolution between the sexes or between hosts and parasites is recognized as a powerful force capable of driving rapid evolutionary changes, which contributes significantly to biodiversity [2], [3]. Since Haldane's [4] proposal on the importance of parasites resulting in genetic adaptations of the host, researchers have acknowledged their potentially important role in speciation [5], [6]. More recently, the concept of cospeciation between parasites and their hosts has received increased attention [7]. The theory of the geographic mosaic of coevolution predicts the diversification of allopatric populations of both host and parasite through three-way interactions between their genotypes and the environment [8].

Consistent with these models, the diversity of parasite species infecting host lineages has been shown to be positively correlated with host species richness in primates [9] and rodents [10]. Some closely-related species also display striking differences in their response to infectious diseases, as exemplified by the difference between humans and chimpanzees (*Pan troglodyte*) in their response to infection by hepatitis B and C, HIV and *Plasmodium falciparum* [11]. Parasitism has also been correlated with increased bacterial host diversity and specialization in microbial communities [2]. Moreover, divergence in MHC genes has been associated with the adaptive radiation of African large barbs from Lake Tana, Ethiopia [12]. Finally, genes showing signs of significant adaptive evolution among rodent and human lineages were found to be related to reproduction, olfaction and immunity [13], [14].

In vertebrates, one of the most important factors determining an individual's susceptibility and resistance (defined here as the likelihood of suffering fitness cost from exposure to a pathogen) to specific parasites is its MHC (major histocompatibility complex) genotype [15]. Classical MHC molecules are cell surface glycoproteins whose primary role is binding and presentation of antigens to T cell receptors as part of a “self/non-self” recognition mechanism representing the first step of adaptive immune response [16]. Antigenic peptides are anchored at specific residues commonly found to be under positive selection and called Antigen Binding Sites (ABS). MHC genes that differ at ABS produce molecules that effectively bind and present different antigens to T cells. This will in turn determine whether or not an effective immune response can be mounted against the infectious agent [16]. The MHC thus initiates the adaptive immune defense which is why these genes are thought to be among the most important targets of the host-parasite coevolutionary arms race [15]. Besides their central role in immunity, MHC molecules are also known for their influence, through olfactory signals, on mate choice and kin recognition, producing either assortative or disassortative associations in various species including humans [17], rodents [18], [19] and fishes [20], [21].

Some of the most spectacular examples of tropical biodiversity are the adaptive radiations of East African cichlid fishes of Lake Victoria, Malawi and Tanganyika [22], [23]. How these hundreds of endemic species evolved from a few founding lineages in a few million years and can coexist within single lakes remains an unresolved issue in evolutionary biology. In Lake Malawi, about 500 endemic species are believed to have diverged from a single lineage within the last 5–7 million years [23]. Particularly intriguing is the apparent lack of clear morphological or ecological adaptive differences among many congeneric species, which are distinguished largely on the basis of colour [24], [25]. Little is known about adaptive genetic differentiation among closely-related species which may lead to evolution of reproductive isolation.

The possibility that host-parasite coevolution has made a significant contribution to East African cichlid diversity has hitherto received scant attention. Nonetheless, several observations suggest that it might be worthy of consideration. Firstly, the most species-rich groups of haplochromines in Lake Victoria and Malawi (i.e. the rock dwelling Mbipi and Mbuna respectively) have highly disjointed and fragmented populations with pronounced genetic structure [26]. This would make them prone to diverge under local coevolutionary dynamics according to the geographic mosaic theory [8]. Secondly, population densities of rock dwelling haplochromines are typically very high, in the order of tens of thousands per km^2^ or more [24], which can favor the spread of infectious diseases [27]. Thirdly, interspecific interactions, like parasitism, are thought to be more intense and complex in tropical ecosystems and might contribute to the higher speciation and specialization observed in these regions [1]. Finally, parasitism has previously been shown to negatively influence male mating success through female choice in a Lake Malawi cichlid of the genus *Mchenga* (formerly *Copadichromis*) [28], and to be negatively correlated with the intensity of carotenoid-based male nuptial coloration and territory size in *Pundamilia nyererei* from Lake Victoria [29].

Recently, it has been demonstrated that olfactory communication plays an essential role in assortative mating between the sympatric Lake Malawi cichlids *Pseudotropheus emmiltos* and *P. fainzilberi* [30]. Divergent natural selection can cause ecological differentiation and speciation simultaneously when its action on ecological traits affects mate choice as a by-product [31]. Considering the documented influence of MHC genotype on odor-mediated mate choice, we hypothesized that odor differences between these species may involve differences in MHC genes accumulated under divergent host-parasite coevolution. In this context, the goal of this study was to document patterns of genetic differentiation between a pair of closely-related sympatric species at MHC class II B, as predicted if host-parasite coevolution has played a role in the divergence of the closely related *P. emmiltos* and *P. fainzilberi.* More, specifically, we predict that such divergence should be particularly prominent when comparing nonsynonymous sequence variation at the exon 2-encoded ABS of different species, as diversifying selection is thought to act most stringently at these sites. Ratios of nonsynonymous to synonymous substitutions (dN/dS) higher than unity were used to identify putative ABS, as these are indicative of positive selection. Comparisons with neutral divergence at non-coding intron or synonymous exon 2 sequences of the same gene were used to assess whether divergent selection had to be invoked to explain observed allelic compositions. We also predicted that divergence in MHC class II should be correlated with detectable differences in parasite communities currently infecting the two species in sympatry. The results show clear evidence of divergent selection at MHC class II B genes between the species. Moreover, we found significant differences in parasite communities collected on each sympatric host species.

## Results

### Positive Selection and Recombination

**Figure 1:**
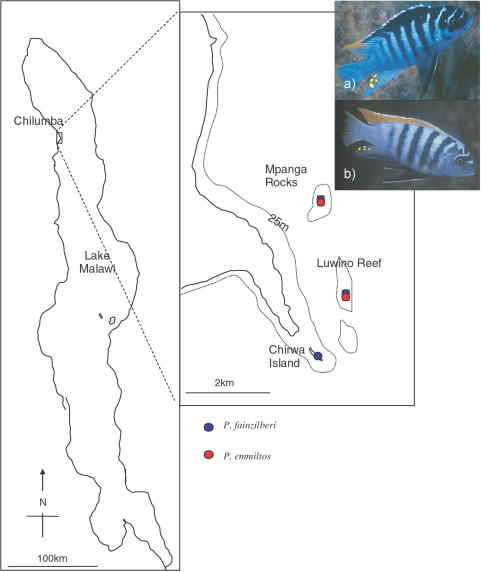
Sampling Locations. Individuals of a) *Pseudotropheus fainzilberi* and b) *P. emmiltos* were collected at Mpanga Rocks, Luwino Reef, and Chirwa Island off the North Western shore of Lake Malawi. The two species are sympatric at Mpanga Rocks and Luwino Reef. Photograhs show males in full nuptial dress and are courtesy of Ad Konings.

MHC class II B intron 1 and exon 2 sequences were obtained from a total of 1022 clones, representing 74 *Pseudotropheus emmiltos* individuals from two populations (Luwino Reef N = 31; Mpanga Rocks N = 43) and 106 *P. fainzilberi* individuals from three populations (Luwino Reef N = 31; Mpanga Rocks N = 36; Chirwa Island N = 37) (Fig. 1; Fig. S1). A mean±standard deviation of 5.74±2.22 clones per individual were sequenced. The final dataset contained 419 individual exon 2 haplotypes (GenBank accession numbers: EF539902-EF540320) and a high level of polymorphism and haplotype diversity comprising 196 distinct exon 2 alleles (172 at the amino acid level) and 164 distinct intron 1 alleles (Fig. S2). The mean number of alleles per individual was 2.31 and 2.29, and ranged from one to five and one to six, for exon 2 and intron 1 respectively (Fig. S1). These data are consistent with Malaga-Trillo et al. [32] that concluded that the number of MHC class II loci per individual varied between one and six (and therefore that the number of alleles varied between one and 12) in *Pseudotropheus zebra*. Although we cannot be certain that we have detected all alleles present in each individual, our sampling nevertheless provide an unbiased and adequate estimate of population allele frequencies.

Likelihood ratio tests comparing selective regimes affecting MHC class II B exon 2 in *P. emmiltos* and *P. fainzilberi* showed that global selection pressures affecting exon 2 were not significantly different between species, neither in terms of the proportion of sites under selection nor in terms of strength of selection (Table S1). Site-by-site analyses revealed three sites (72, 85 and 87) evolving under different selective pressures (with p<0.05) between the two species along internal branches and three sites (50, 74 and 87) at the tip of the phylogeny. However, these results were not significant after Bonferroni correction. The identification of codons under significant positive selection was therefore conducted on sequences pooled from both species.

**Figure 2:**
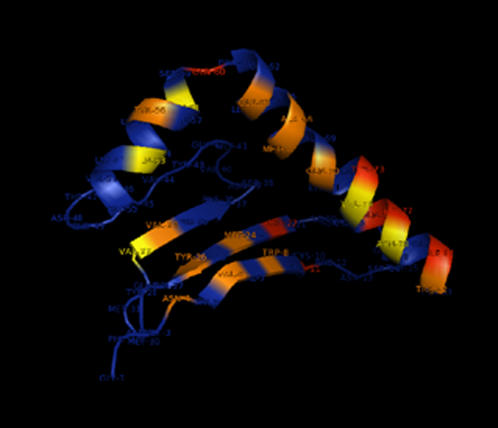
Three-Dimensional Model of the β_1_ Domain of Cichlid MHC Class II. The tertiary structure prediction was based on Psemmil_m58_c exon 2 sequence (Accession number: EF540049) of 41% homology with mouse MHC sequence gi|13399459 in the Protein Data Bank (http://www.pdb.org/pdb/home/home.do) using the 3D-jigsaw server v.2.0 (http://www.bmm.icnet.uk/~3djigsaw/). The graphical representation was created using the program Pymol v.0.99. Amino acid residues under significant positive selection in *Pseudotropheus fainzilberi* and *P. emmiltos* and corresponding to peptide binding sites in humans are highlighted in orange. Residues shown in red were under significant positive selection in *P. fainzilberi* and *P. emmiltos* but do not correspond with peptide binding sites in humans. Residues in yellow are human peptide binding sites that were not found to evolve under positive selection in cichlids. The 84 amplified exon 2 codons are numbered 1-84 on the graph. Those correspond to codons 6-89 of the mature protein.

**Figure 3:**
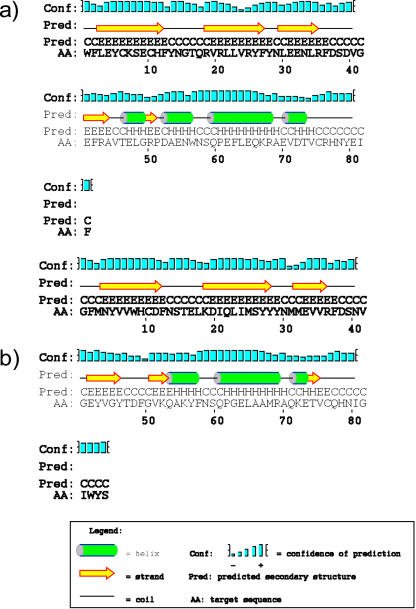
Comparison of Secondary Structure of Mouse and Cichlid MHC Class II Exon 2. Structure predictions were obtained from the PSIPRED server (http://bioinf.cs.ucl.ac.uk/psipred/psiform.html) for a) mouse sequence gi:13399459 and b) cichlid sequence Psemmil_m58_c (Accession number: EF540049). Mammalian and fish secondary structures are similar except for an additional short alpha-helix at position 46–49 predicted for the mouse sequence, and an additional short strand at position 74 predicted for the cichlid sequence.

Constraining dN≤dS revealed clear evidence of global positive selection in exon 2 since the likelihood of this model was significantly lower than the one allowing dN>dS (LRT = 648.554; d.f. = 3; p<0.001). The bivariate REL analysis identified four dN/dS rate categories across exon 2, with mean rate estimates of: 0.43, 1.11, 2.40 and 5.75. We considered the two lower rate categories as representing purifying selection and (nearly) neutral evolution respectively, and the two higher rate categories as indicating positive selection. A total of 19 out of 84 codons had an empirical Bayesian posterior probability greater than 0.95 of belonging to one of the two positive selection rate categories (Table S2). Of these, 12 corresponded with human ABS identified from crystallography [33] (Fig. 2), suggesting that although the functional structure of mammalian and fish MHC class II molecules are approximately similar (Fig. 3; Fig. S3), there appear to be noteworthy differences in the precise position of ABS codons. In mammals and fish, 59 and 58% respectively of MHC class II B ABS are found in the alpha-helices or in coils between adjacent helices forming the edges of the peptide binding groove. A similar proportion (52%) of positively selected sites was found in the alpha-helices of the peptide binding groove of non-classical mhc class Ib genes thought to be involved in pheromone detection in rodents [34].

We compared intron 1 and exon 2 mean genetic distances (*d* and *dS* respectively) within 14 functional clades defined using these positively selected sites (from an amino acid p-distance neighbor-joining phylogeny, Fig. S4). Significantly different mean genetic distances between intron 1 and exon 2 were detected in at least eight clades, which is evidence for intron-exon recombination [35] (Table S3). In three of these, mean *d* at intron 1 was significantly lower than mean *dS* at exon 2, suggesting the action of within-locus recombination and balancing selection on functional exon 2 alleles [35]. The remaining five cases had mean *d* at intron 1 higher than mean *d*S at exon 2, suggesting inter-locus recombination [36]. However, among those eight clades, only clade 14 had bootstrap support higher than 60% and thus results from the other seven clades must be viewed with great caution. Nevertheless, a single breakpoint recombination analysis [37] revealed that the most likely breakpoint was at nucleotide position 231. Including this recombination breakpoint greatly improved the phylogenetic model of intron 1 and exon 2 evolution (ΔAICc = 13001.0; ΔBIC = 3667.85). This position is located only 15 nucleotides upstream from the intron 1-exon 2 junction of the alignment, corroborating the scenario of recombination between intron 1 and exon 2.

### Functional Divergence

**Figure 4:**
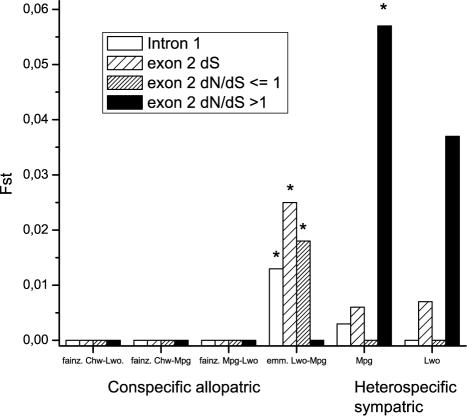
Comparison of Genetic Divergence Between Heterospecific Sympatric Populations and Conspecific Allopatric Populations. Fst values (HudSon et al. 1992) from pairwise comparisons of conspecific allopatric populations (Mpanga Rocks (Mpg), Luwino Reef (Lwo), and Chirwa Island (Chw)) and between heterospecific sympatric populations of *P. fainzilberi* (fainz.), and *P. emmiltos* (emm.). Neutral Fst estimates were obtained from intron 1 p-distances, modified Nei and Gojobori synonymous distances at exon 2, and from amino acid EX distance at exon 2 sites outside the putative ABS region (dN/dS≤1) and functional Fst were obtained from EX distance at putative exon 2 ABS (dN/dS>1). Negative Fst estimates were forced to zero. Evidence of significant genetic divergence between pairs of allopatric populations was found only for neutral Fst estimates between *P. emmiltos* populations from Luwino Reef and Mpanga Rocks. Evidence of significant genetic divergence between pairs of *P. fainzilberi* and *P. emmiltos* sympatric populations was found only at putative ABS at Mpanga Rocks. ^*^Significantly different from zero (p<0.05; 200 permutations)

The association index (AI) value calculated from the intron 1 phylogeny (0.69) was higher than that computed from the exon 2 phylogeny (0.61), suggesting that phylogenetic divergence was higher at exon 2 than at non-coding intron 1 (Table 1). Similarly, genetic differentiation measured by Fst value based on overall mean pairwise genetic distances was higher at exon 2 putative ABS amino acid sites (Fst = 0.097; p<0.01) than at neutrally evolving intron 1 (Fst = 0.021; p<0.01) or synonymous exon 2 alleles (Fst = 0.021; p<0.01). Both findings are contrary to what is predicted from a scenario of balancing selection with no divergent selection at exon 2. In addition, within exon 2, Fst was higher for positively selected putative ABS (Fst = 0.097; p<0.01) than for sites outside this region (Fst = 0.016; p = 0.015) (Table 1). Moreover, higher divergence at putative ABS compared to neutral alleles was observed for both heterospecific sympatric comparisons, but for none of the conspecific allopatric comparisons where functional Fst estimates at ABS were all negative and therefore technically equal to zero (Fig. 4).

**Table 1:** Neutral and functional genetic differentiation estimates between *Pseudotropheus fainzilberi* and *P. emmiltos* samples.

	Fst^1^	AI^2^
Intron 1 nucleotide p-distance	0.021†	
Exon 2 dS*	0.021†	
Exon 2 (dN/dS≤1 sites) EX**	0.016††	
Exon 2 (dN/dS>1 sites) EX**	0.097†	
Exon 2 Kimura 2-parameters		0.612‡
Intron 1 Kimura 2-parameters		0.690‡

Neutral Fst values were calculated from intron 1, exon 2 synonymous alleles (exon 2 dS) and the 65 exon 2 amino acid sites evolving under a mixture of purifying and nearly neutral evolution (dN/dS≤1 sites). Functional Fst estimate was obtained from the 19 amino acid exon 2 putative ABS (dN/dS>1 sites). Association indices (AI) were obtained for intron 1 and for exon 2 phylogenies from neighbor-joining trees using Kimura 2-parameters distances and a *Salmo salar* outgroup sequence (gi:57335063) in the HYPHY package. AI values reflect the level of phylogenetic compartmentalization and correspond to the mean ratio of the sum over all nodes of the association values *d = (1-f)/2^n−1^* from 100 bootstrapped tree of the test sequences on species-reassigned control. Lower values reflect higher divergence of samples.

1 (Hudson et al. 1992); ^2^ (Wang et al. 2001); †significantly different from zero (p<0.01) from 200 permutations; ^††^significantly different from zero (p = 0.015) from 200 permutations; ‡significantly different from zero (P<0.01) from 100 bootstrap tree replicates; * *dS* calculated using the modified Nei and Gojobori method with transition/transversion = 1.26 estimated from the data; ** Experimental exchangeability distance (EX) for amino acid [81] estimated by maximum likelihood.

Finally, there were 31 shared and 23 private alleles at intron 1 between *P. fainzilberi* and *P. emmiltos*, compared to 22 shared and 41 private alleles at exon 2 amino acid sequences. These raw proportions were significantly different (two-tailed Fisher's Exact Test: p = 0.017). There was no significant difference in allele frequency distributions between exon 2 and intron 1 (Fig. S5), (mean *f* intron 1: 0.015, mean *f* exon 2: 0.013, Mann-Whitney U = 1558.000, p = 0.437; intron 1 σ^2^: 0.246, exon 2 σ^2^: 0.227; Levene's test F = 0.435, p = 0.511). There was, however, a highly significant difference in shared and private allele composition as measured by *Dc* (Table 2). This difference was significant between intron 1 and exon 2 alleles when considering the whole exon 2 sequence, and between intron 1 and exon 2 alleles at the 19 positively selected putative ABS, but not between intron 1 and the 65 exon 2 residues outside this region (Table 2).

**Table 2:** Proportions of private and shared alleles between *Pseudotropheus fainzilberi* and *P. emmiltos* at MHC class II B intron 1 alleles and exon 2 amino acid alleles.

	N shared	N private	Mean *f* private	Mean *f* shared	Mean *Dc*	Median *Dc*	Rank sum	Mann-Whitney U	2*one-sided exact p
Intron 1	31	23	0.007	0.020	−0.563	−0.981	2773.500		
dN/dS ≤1	26	32	0.008	0.023	−0.437	0.005	3554.500	1288.500	0.106
Intron 1	31	23	0.007	0.020	−0.563	−0.981	2760.500		
dN/dS>1	23	42	0.007	0.025	−0.343	0.005	4379.500	1275.500	**0.010**
Intron 1	31	23	0.007	0.020	−0.563	−0.981	2590.500		
Exon 2	22	41	0.007	0.023	−0.339	0.005	4312.500	1105.500	**0.001**

Distinctiveness scores *Dc* between intron 1 alleles and the whole sequence of exon 2 amino acid alleles (Exon 2), between intron 1 alleles and positively selected putative ABS amino acid sequences (dN/dS>1), and between intron 1 alleles and exon 2 amino acid alleles outside the putative ABS region (dN/dS≤1), were compared using Mann-Whitney *U* tests. Significant p-values (α = 0.05) are in bold.

### Comparison of Parasite Communities

Sixteen categories of parasites were identified in both species and their abundance recorded on each fish (Table 3). A Principal Component Analysis on the dataset [38] returned four factors explaining 84% of the original variance (Table 3). PC1 represented mainly a negative correlation between infection by acanthocephalans and stomach cysts versus infection by muscle-encysted digenean metacercaria, PC2 between infection by muscle-encysted digenean metacercaria and by stomach cysts, and PC3 between infection by ciliate *Trichodina* sp. and gill parasitic copepod sp. 3 versus infection by acanthocephalans. Finally, PC4 largely represented a contrast between infection by gill parasitic copepod sp. 3 and ciliate *Trichodina* sp.

**Table 3:** Parasite infections of *Pseudotropheus emmiltos* and *P. fainzilberi*: eigenvectors of the first four factors explaining 84% of the original variance returned from a principal component analysis on incidence and abundance of 16 types of parasites transformed according to equation 12 in Legendre and Gallagher [38].

	PC1	PC2	PC3	PC4
Muscle-encysted digenean metacercaria	0.474	0.704	−0.303	−0.144
Microsporidians	−0.030	0.021	−0.004	0.063
Gill infecting monogeneans (*Cichlidogyrus* sp.)	0.005	0.009	0.023	−0.025
Skin and gill infecting monogeneans (*Gyrodactylus* spp.)	−0.041	−0.020	−0.049	−0.008
Glochidia larvae	0.020	−0.008	−0.013	−0.004
Eye infecting digenean sp.	0.057	0.028	0.136	0.364
Gill infecting copepod sp. 1	−0.022	0.020	−0.020	0.055
Gill infecting copepod sp. 2	0.038	−0.016	0.095	0.150
Gill infecting copepod sp. 3	−0.108	0.050	0.418	0.568
Acanthocephalans	−0.636	−0.121	−0.522	−0.133
Plagiorchid digenean (*Astriotrema turnerei*)	−0.013	−0.023	−0.048	−0.028
Hepatic cyst sp. 1	0.007	−0.010	−0.007	−0.004
Hepatic cyst sp. 2	−0.039	−0.005	−0.038	−0.032
*Trichodina* sp.	−0.102	0.018	0.631	−0.682
Bladder infecting monogeneans (*Urogyrus* sp.)	−0.035	0.011	0.036	0.046
Stomach cyst	0.581	−0.696	−0.162	−0.082
% total variance	43.278	21.000	11.763	8.000
% cumulative variance	43.278	64.278	76.041	84.041

A multivariate linear model of the relationship between these first four factors representing infecting parasite communities and host species, host sex, host standard length and collection site returned R^2^ values of 0.754, 0.473, 0.178 and 0.010 for PC1 to PC4 respectively. Overall, 26% of the variance in these four factors was explained by variation among collection sites, while host standard length, host sex and host species accounted for 3.6, 3.3, and 1.8% of the variance, respectively (Table 4). There were significant differences between *P. fainzilberi* and *P. emmiltos* in PC1 and PC3 (Table 4). Least square means for PC1 and PC3 were 0.263 and −0.372 for *P. emmiltos* and 0.027 and 0.067 for *P. fainzilberi* respectively. Hence, *P. emmiltos* tended to have more stomach cysts and muscle encysted digenean metacercaria relative to acanthocephalan infections (PC1) than *P. fainzilberi*. Conversely, *P. emmiltos* had less trichodinids and gill infecting parasitic copepod sp 3 relative to acanthocephalan infection (PC3) than *P. fainzilberi*.

**Table 4:** Sum of squares (SS) and degrees of freedom (df) of a multivariate general linear model testing the effect of host species, collection site, host sex, and host standard length on four principal component analysis factors (PC1-PC4) representing 84% of the original variance of the incidence and prevalence of 16 different types of parasites.

		PC1		PC2		PC3		PC4		Total	% Variance explained
	df	SS	Coeff. 95% CI	SS	Coeff. 95% CI	SS	Coeff. 95% CI	SS	Coeff. 95% CI	SS	
Intercept	1	1.024	**−2.127–(−0.133)**	5.639	**−4.367–(−1.388)**	3.048	−0.076–4.138	0.002	−1.866–2.007	9.713	0.029
Host species	1	0.925	**0.023–0.241**	1.130	−0.002–0.285	3.194	**−0.399–(−0.03)**	0.604	−0.339–0.144	5.853	0.018
Collection site	2	55.719	**−1.016–(−0.811);**	20.359	**0.207–0.512;**	8.056	**−0.018–0.447;**	0.366	−0.196–0.287;	84.500	0.256
			**0.115–0.253**		**0.286–0.503**		**−0.358–(−0.114)**		−0.207–0.100		
Host sex	1	2.477	**−0.351–(−0.121)**	8.067	**−0.571–(−0.250)**	0.254	−0.317–0.173	0.046	−0.284–0.235	10.844	0.033
Host standard length	1	1.289	**0.004–0.029**	6.952	**0.021–0.058**	3.515	**−0.053–(−0.001)**	0.000	−0.026–0.022	11.756	0.036
Error	75	19.666		42.157		65.801		79.235		206.859	0.628
										329.525	

95% confidence intervals for the coefficients of the model (Coeff. 95% CI) are the 5% and 95% bias corrected and accelerated (BCa) percentiles of 1000 bootstrap replicates of the original data. Significant effects correspond to CI excluding zero and are marked in bold.

## Discussion

Antagonistic host-parasite interactions involving coevolutionary responses have the potential to produce rapid evolutionary changes [3]. Genes directly implicated in these interactions are often polymorphic [39] and may diverge quickly if there are differences in selective pressures among populations [8]. Such defense genes are likely candidates to show the first signs of genetic divergence in recently-speciated taxa. Whereas the importance of divergence in traits related to defense against parasites and other enemies (e.g. phytophagous insects) has long been recognized in studies of plant radiations, it has remained largely ignored in the study of adaptive radiations of animals [40]. Here, we tested the hypothesis that two recently diverged species experienced divergent selection at important immunological genes of the MHC class II. Furthermore, we also tested for possible differentiation in parasite communities infecting the species in sympatry, which we hypothesized could be a driver of divergent evolution at the MHC.

Evidence for strong balancing and positive selection on MHC class II loci was manifested by the high polymorphism and haplotype diversity and a clear signal of positive selection across the 84 codons of exon 2. Differentiation levels expressed in the association index (AI) and Fst, as well as proportion of shared and private alleles for exon 2 of MHC class II B were contrasted with neutral divergence at intron 1 and synonymous exon 2 polymorphisms. These comparisons revealed that the observed patterns of functional allelic composition of the two species cannot be explained by divergence due to drift alone.

Four arguments support the contention that diversifying selection augmented genetic differentiation of parts of the MHC genes between the two cichlid species. Firstly, without divergent selection, genes under balancing selection are expected to show less differentiation between populations than neutral loci [41], [42]. However, despite recombination between intron 1 and exon 2, divergence estimated by AI values was higher for the exon 2 phylogeny than for the intron 1 phylogeny. Secondly, neutral Fst from intron 1, exon 2 dS, or sites with dN/dS≤1 in exon 2 was about 2%, while this value was almost five times higher in putative ABS. Thirdly, this pattern of higher divergence at putative ABS was observed between both heterospecific sympatric population pairs but for none of the allopatric conspecific population pairs, suggesting that this is a species-specific adaptive divergence that persists despite potential gene flow between species in sympatry. Finally, the significantly higher proportion of private alleles at exon 2 amino acid sequences compared with non-coding intron 1, provides strong additional evidence that despite close physical linkage between intron and exon sequences, divergent selection contributed to the shaping of the functional allelic composition of the species.

Adaptive divergence at immune system genes is likely to be correlated with differences in resistance and susceptibility to parasites. Our results are consistent with this prediction as there were significant, albeit relatively small, interspecific differences for PC1 and PC3 while controlling for host sex, standard length and collection site. Apart from acanthocephalans and unidentified stomach cysts, the other parasites represented by PC1 and PC3 (namely digenean metacercaria, trichodinids and gill infecting parasitic copepod) are known to actively infect the fish from the water column. Acanthocephalans are normally acquired via the food chain. There is therefore two distinct modes of transmission involved. Thus we cannot rule out the possibility that species differences in susceptibility stems from differences in microhabitat or diet rather than from genetic differences [43]. However, there is substantial overlap in microhabitat and both species feed by combing attached filamentous algae with their specialized bi- and tricuspid teeth [24]. In addition, at sampling locations, males of both species have overlapping territories which they use for foraging.

A substantial amount of variance (26%) in infecting parasite communities was explained by variation among collection sites. Local variation in abundance of specific parasites can be due to transient epidemics or alternatively, to more stable variation in population densities caused by habitat or environmental differences. The latter case provides the raw material for coevolutionary divergence in genes involved in parasite resistance such as MHC between allopatric populations exposed to different parasite environments [8]. Upon secondary contact, such differences accumulated in allopatry could be manifested by distinct infection patterns of the species such as those detected here. Alternatively, initial divergence among allopatric populations in trophic niche could have lead to different levels of exposures to parasites transmitted via the food chain such as acanthocephalans, which in turn may select for different MHC alleles. These processes of differentiation could be particularly important in species with pronounced genetic structure such as rock-dwelling haplochromines. Divergence in immune system does not imply detectable divergence in morphology and may therefore be overlooked in many animal systems. Nevertheless, divergence in immunity could have important consequences on foraging or reproductive behaviors that may in turn promote speciation or species coexistence. The phenomenon could be more widespread in animal radiations than currently appreciated. For example, divergence in MHC class II has been observed in the radiation of large barbs (cyprinid fishes) in Ethiopia's Lake Tana [12]. In the cyprinodont *Fundulus heteroclitus*, divergent selection in MHC class II among populations was inferred from population specific amino acid replacement at putative ABS [44].

Identifying adaptive genetic differentiation between closely related species is an important step toward understanding speciation processes. Unfortunately, this critical information has been lacking in the case of African haplochromine cichlids. The impressive species diversity and endemism of cichlid species assemblages from Lake Malawi, Victoria and Tanganyika have established the systems as some of the most important models in evolutionary biology [45]. Part of this fascination comes from the surprising ecological and morphological similarity of congeners, which led some to suggest that natural selection had little importance in recent speciation events [46], [22]. The results presented here provide one of the first evidence of adaptive genetic evolution in African cichlids from the African Great Lake radiations [see also 47], [48]. We hypothesize that this adaptive differentiation may have fuelled the speciation process as a by-product by augmenting MHC-mediated mate choice signals. Natural selection from host-parasite coevolution may thus have played a more important role in recent speciation of endemic African cichlid lineages than suggested by morphological similarities.

Sexual selection has long been regarded as an important factor in haplochromine speciation [49]–[51] while the importance of parasitism in sexual selection and mate choice has been discussed extensively [52]–[59]. Several cases of assortative MHC-based mate choice have been documented. Thus, MHC genotypes represent an ecological trait whose divergence by natural selection may directly produce reproductive isolation as a by-product. For instance, threespine stickleback females prefer to mate with males having an intermediate rather than maximal number of MHC class II alleles different from those the females possess [60]. Female preference for intermediate MHC similarity has also been found in humans [17]. Malagasy giant jumping rat (*Hypogeomys antimena*) females preferred males with MHC genotypes more similar to themselves [19]. Possible causes of mate preferences aimed at limiting offsprings' MHC diversity include detrimental effect of excessive MHC diversity caused by: negative thymic selection of T cell lines [61], [62], cross-reactive autoimmune diseases [62] and reduced concentration of specific MHC-peptide ligands on the surface of antigen presenting cells for effective T cell activation [63]. We therefore speculate that multilocus MHC genotype compatibility may exclude matings between *P. fainzilberi* and *P. emmiltos* through olfactory signals part of a mate choice rule including some limits on MHC dissimilarity of mates. This would represent a case of “one-allele” speciation mechanism [64] as the same mate choice alleles in both species would reduce gene flow. Behavioral studies on the precise influence of MHC genotype on mate choice in these two species may elucidate the role that this divergence has played in the evolution of reproductive isolation.

## Materials and Methods

### Study Sites and Field Sampling

*Pseudotropheus emmiltos* and *P. fainzilberi* are among the 17–44 currently recognized species of the *P. zebra* species complex (subgenus *Maylandia*), members of which are found all around in Lake Malawi [23]. The two species are sympatric at Mpanga Rock and Luwino Reef off the north western coast (Fig. 1), the only two sites where *P. emmiltos* is known to occur [65]. *P. fainzilberi* is found at several other localities both north and south of the zone of sympatry. Thus, the range of *P. emmiltos* is nested within the range of *P. fainzilberi*. Mitochondrial DNA analyses using the model of isolation with migration implemented in the program IM [66] revealed that the two species diverged around 30–45 000 years ago and that the most probable speciation scenario was one of allopatric divergence followed by secondary contact (Turner et al. in prep.). The most noticeable morphological difference between the taxa is the color of the dorsal fin, which is orange in *P. emmiltos* and blue with a black longitudinal stripe in *P. fainzilberi* (Fig. 1). The two species are behaviorally reproductively isolated and olfactory signals are involved in mate discrimination [30]. Fifty individuals of each species where caught at Mpanga Rocks and Luwino Reef by SCUBA divers using monofilament gill nets. Fifty individuals of *P. fainzilberi* were also captured at Chirwa Island, two kilometers south of the zone of sympatry (Fig. 1).

### Cloning and DNA Sequencing

MHC class II molecules are expressed on the surface of antigen presenting cells and bind antigenic peptides in a cleft formed by the α_1_ and β_1_ domains in which peptides are anchored in pockets of specific residues (ABS). Bound peptides are then presented to CD4+ T cells. Sequence polymorphisms at these sites are believed to result from positive and balancing selection [67], [68]. Intron 1 along with 252 bp of exon 2 (codons 6-89 of the mature protein), coding for the β_1_ domain of MHC class II genes [69] were PCR amplified using the degenerate primer pair TU377-TU383 [32], which amplifies up to eight expressed putative loci in African cichlids according to comparisons of individual haplotypes obtained from genomic and cDNA clones [32; Blais et al. unpublished results]. PCR conditions consisted of 30 cycles of 30 sec at 95°, 60 sec at 56°, 60 sec at 72° in a 20 µl reaction volume containing 0.25mM of each dNTPs, 2 µl of 10× reaction buffer, 2 mM MgCl_2_, 0.5U of Taq DNA polymerase, and 25 ρmol of each primer.

PCR products were purified by ExoSAP-IT (GE healthcare, Chalfont St. Giles, U.K.) following manufacturer's instructions, ligated into pGEM®-T vector (Promega, Madison, WI, USA) and cloned using JM109 Competent Cells (Promega, Madison, WI, USA). Between eight and 30 recombinant clones per individual were amplified with primers OMNI (5′-ACA GGA AAC AGC TAT GAC CAT GAT-3′) and UNI (5′-CGA CGT TGT AAA ACG AGG CCA GT-3′) under the same PCR conditions described above, and sized on 1.5% agarose gel. Between four and 23 clones containing inserts of expected size (∼320–500 bp depending on intron 1 length) per individual were purified with ExoSAP-IT (GE healthcare, Chalfont St. Giles, U.K.), sequenced using Bigdye 2.0 dye terminator kit (Applied Biosystems, Foster City, CA, USA), cleaned with Sephadex™ and analyzed on an ABI 3100 DNA Analyzer (Applied Biosystems, Foster City, CA, USA). Sequence alignments produced by ClustalX v.1.83 [70] were examined to detect false allelic variants due to PCR errors by comparing sequences within and among individuals. First, sequence differences of less than 3 bp when compared to multiple identical sequence replicates from different clones within individuals were considered to be PCR or cloning artifacts and removed from the alignment. Second, only sequences found in at least two independent PCR (within or among individuals) were used for subsequent analyses [42].

### Identification of ABS

There is at present no available information from X-ray diffraction or NMR analyses about the three-dimensional structure of non-mammalian MHC. It is therefore impossible to identify with certainty which sites are involved in peptide binding. In order to identify putative antigen binding sites (ABS) within the peptide binding cleft involved in the coevolutionary arms race, we determined which codons were evolving under positive selection, i.e. having a nonsynonymous/synonymous substitution ratio (dN/dS) significantly higher than unity. We also compared the location of these with mammalian ABS identified from crystallography. The following and all subsequent sequence analyses were conducted using the HYPHY package and programming language running on a 32 processors SGI Altix3700 computer at the Bioinformatics and Computational Biology Centre of University Laval, under a maximum likelihood framework, unless otherwise stated .

### Recombination

According to Kosakovsky Pond et al. [37] and as implemented by SingleBreakpointRecomb.bf in HYPHY, we determined the most likely recombination breakpoint by computing the likelihood of neighbor-joining phylogenies [71] for the entire intron-exon alignment and for models fitting branch length independently for partition blocks on each side of a single break point located at each nucleotide position sequentially. Akaike information criterion with second-order correction (AICc) and Bayesian information criterion (BIC) were used to determine which was the most likely recombination breakpoint in the sequence and if including recombination improved the fit of the phylogenetic model (SingleBreakpointRecomb.bf in HYPHY). Using a method assuming a single breakpoint was shown [37] to perform as well or better than 14 different recombination detection methods evaluated by Posada and Crandall [72] and is particularly well suited for relatively short alignments like the one studied here where one is interested in evaluating the likelihood of recombination at a specific location along the sequence.

### Selection

In the next sections, we explain the methodology used to examine evidence for different selection pressures: (1) positive selection, (2) balancing selection and (3) divergent selection. Here, we define positive selection as selection for improved recognition of parasites. Positive selection pressure favors nonsynonymous substitutions particularly in the ABS.

Balancing selection is thought to be the dominant force maintaining polymorphism in classical MHC genes [67], [73], [74]. Overdominance and negative frequency dependent selection are types of balancing selection that operate within populations. In the absence of divergent selection, balancing selection is expected to prevent or delay population divergence [41], and some empirical studies suggested that genetic differentiation between populations is lower for functional MHC alleles than for neutrally evolving loci [42]. Balancing selection thus maintains a high level of polymorphism within populations but reduces genetic divergence between populations.

Finally, divergent selection occurs when different selection pressures act between populations, resulting in higher genetic differentiation, particularly at dN of ABS of allopatric populations. If populations differ in local (positive) selective pressures, divergent selection may maintain different sets of alleles and result in increased differentiation [44]. Divergent selection could thus counteract the affect of balancing selection and increase the extent of population differentiation at a level above that expected for neutral genes [reviewed in 15].

### Positive Selection

Before determining which individual codons were under positive selection, we first verified whether the selective regimes affecting each species were the same in terms of proportion of sites under selection and the strength of selection. We therefore constrained the substitution rate parameters by setting it equal for both species, and compared the fit of this model with models that could use different parameter setting for each species. The fit of the models were compared using likelihood ratio tests (LRT) with CompareDistribution.bf in HYPHY. A codon-based site-by-site fixed effect likelihood (FEL) analysis comparing selective pressures between the two species along internal branches and leaves of the phylogenetic tree was also performed (ComparesSelectivePressure.bf in HYPHY) [75]. This analysis tests the null hypothesis that the ratio of nonsynonymous to synonymous substitutions is significantly different between the two species for each codon site using likelihood ratio tests.

Positive selection acting on exon 2 was tested by comparing a random effect likelihood (REL) dual model [76] where both dN and dS are free to vary and are drawn from a general discrete distribution with N = 4 rate classes but where dN is constrained to be≤dS, with a model where dN is free to be>dS [77]. A likelihood ratio test (LRT) based on the Chi-square distribution with N–1 degrees of freedom was used to determine whether allowing dN>dS significantly improved the fit of the model. Individual codons evolving under significant positive selection were identified using a REL method where both dN and dS are free to vary and were drawn from a bivariate general discrete distribution with four dN and four dS rate categories (dNdSBivariateRates.bf in HYPHY). The model fits the parameters of this bivariate general discrete distribution of rates across the sequences given the phylogeny. Positive selection at specific codons is inferred for sites having an empirical Bayesian posterior probability≥0.95 to be in one of the dN/dS>1 categories. The empirical Bayesian approach is based on the maximum likelihood estimates of the rate distribution parameters [78]. The results were then compared with sites of the cichlid MHC sequence corresponding to human ABS deduced from three-dimensional information.

### Balancing Selection

Following Hughes [35], evidence of both balancing selection and recombination between exon 2 and intron 1 in each species was evaluated by comparing mean genetic distance (*d*) among intron 1 alleles and mean synonymous genetic distance (*dS*) at functional exon 2 alleles within functional clades defined using putative ABS identified by the method outlined above. Recombination between introns and exons creates incongruence between intron and exon phylogenies. This translates into significantly lower mean genetic distance among intron alleles than exon 2 alleles within exon 2 functional clades, assuming recombination occurs within locus and that balancing selection acts on exon 2 functional allelic variants [35]. Indeed, balancing selection on the ABS can maintain synonymous sequence variation in exon 2 due to genetic hitchhiking, while recombination causes intron 1 to loose polymorphism by genetic drift. On the other hand, higher genetic distance at introns than at exon 2 within functional clades is expected when recombination occurs predominantly between loci [36].

### Divergent Selection

We tested for the presence of divergent selection using three different approaches. First, the level of phylogenetic compartmentalization at non coding intron 1 was compared with that observed at exon 2 using the association index (AI) developed by Wang et al. [79] and implemented in HYPHY. An association value, *d*, for the tree is calculated by summation of *d*-values from each node, according to *d* = (1–*f*)/2*^n^* ^−1^, where *n* is the number of sequences below the node and *f* is the frequency of sequences from the most common species in the clade. Values of *d* expected from the null hypothesis (i.e. no phylogenetic grouping) were calculated by random reassignment of the sequences to the different species. The association index (AI) represents the mean ratio of 100 bootstrap replicates of the association value calculated from the test sequences to those of species-reassigned controls from 200 permutations [79]. Smaller AI values indicate higher divergence of samples (i.e. more homogenous clades). Smaller AI values are predicted for intron 1 phylogeny than for exon 2 phylogeny under a pure scenario of balancing selection acting on ABS in exon 2 involving intron-exon recombination [35]. By contrast, divergent selection between species at exon 2 is predicted to result in relatively high AI value for intron 1.

Secondly, estimates of Fst based on pairwise genetic distances [80] were contrasted between neutral (non-coding or synonymous alleles) and functional alleles (i.e. alleles differing in putative ABS amino acid residues). Divergent selection can be inferred in cases where Fst estimates from functional alleles exceed those obtained from neutrally evolving alleles. We calculated neutral Fst estimates using nucleotide p-distance for intron 1, as well as modified Nei and Gojobori *dS* at exon 2. We then computed functional divergence at exon 2 using the amino acid experimental exchangeability distance (EX) [81] estimated by maximum likelihood in HYPHY, for the 65 sites with dN/dS≤1 and for the 19 sites with dN/dS>1 separately. The EX distance is based on experimental assays of the severity-of-effect of amino acid substitutions on protein function and was specifically designed to reflect functional differences in amino acid sequences while being free of mutational bias. Adaptive divergence between species was first tested using Fst values calculated from sequences of all individuals. In order to test whether observed divergence patterns were species specific and thus could be associated with putative speciation processes, we also compared neutral and functional Fst estimates obtained from the two heterospecific sympatric pairs of populations with those obtained from all pairwise comparisons of allopatric conspecific populations.

Third, we compared haplotype compositions of exon 2 amino acid alleles with non-coding intron 1 alleles. In genes such as classical MHC where haplotype diversity is typically very high, most haplotypes may appear only once in the sample and methods based on haplotype frequencies, such as Gst [82], have little or no power [83], [84]. To circumvent this problem, we contrasted the relative proportion of shared and private alleles at intron 1 and exon 2 amino acid sequences. Divergent selection for alternative sets of functional exon 2 alleles predicts that the proportion of private alleles should be higher for exon 2 amino acid sequences than for non coding intron 1 sequences. Alleles that were observed only once were omitted as these cannot be shared by both species. We then tallied the number of ‘private alleles’ (i.e. alleles that were observed at least twice in only one of the two species) and used Fisher's exact test on observed proportions of shared and private alleles at intron 1 and exon 2. We also quantified the relative proportion of private and shared alleles by assigning allelic distinctness scores *Dc* to each allele defined as allele frequency *f* in the case of private alleles, and as-(1-*f_s_*) in the case of shared alleles, where *f_s_* is the frequency of the allele in the species where it is most common. Thus, according to this index, *Dc* is bounded by −1 and 1 (private alleles have positive scores and shared alleles have negative scores), and more weight is given to common private alleles and to rare shared alleles, reflecting the likelihood of sampling each category. We tested for a significant difference between intron 1 and exon 2 in the composition of private and shared alleles as measured by *Dc* using the non-parametric Mann-Whitney *U* test.

### Parasite Screening and Analysis

Heterospecific populations in sympatry were predicted to show different parasite communities due to independent host-parasite coevolution (i.e. a prediction consistent with divergent selection). We therefore dissected a total of 81 fish captured at Mpanga Rocks (*P. fainzilberi* N = 16; *P. emmiltos* N = 18), Luwino reef (*P. fainzilberi* N = 18; *P. emmiltos* N = 17) and Chirwa Island (*P. fainzilberi* N = 12) by SCUBA diving using gill nets in September 2005. The fish were examined for all eukaryote parasites within 24 h of collection. Parasites incidence and abundance were recorded by killing the fish in an overdose of 0.2% MS222 and examining the dissected organs under a stereo-microscope with cold light illumination. The resulting parasite community dataset was subjected to a linear transformation according to 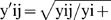, where y_ij_ denotes the observation for parasite type j, on host i and y_i+ _is the sum of observations for host i [eq. 12 in 38]. As a result of this transformation, a principal component analysis (PCA) performed on the transformed data is based on Hellinger distances, suitable for community data comparisons, rather than Euclidian distances [38]. Thus, to reduce the dimensionality of the data, the minimum number of factors required to explain at least 80% of the original variance were retained from a PCA on the transformed dataset. These factors were then used in a multivariate general linear model to test for differences in relative abundance and composition of parasite communities infecting the two host species. The model included host species as categorical predictor, as well as collection site, host sex and host standard length as additional categorical and continuous covariates. Confidence intervals around model coefficients were obtained from 1000 bootstrap replicates of the original dataset in S-Plus.

## Supporting Information

Figure S1 List of intron 1 and exon 2 alleles found in each sampled individual. Each individual fish identifier is given in the leftmost column followed by its species, population and its alleles. Alleles are numbered 1-164 and 1-196 for intron 1 and exon 2 alleles respectively. Individual sequences were deposited in the GenBank database under accession number EF539902-EF540320.
(0.09 MB XLS)

Figure S2 GenBank accession numbers for intron 1 and exon 2 alleles. Accession numbers for sequences corresponding to intron 1 and exon 2 alleles as numbered and listed in figure S1.
(0.10 MB XLS)

Figure S3 Structural Alignment of Cichlid MHC Class II Exon 2 Amino Acid Sequences to Protein Data Bases. The HHpred server (http://toolkit.tuebingen.mpg.de/hhpred) using a profil hidden Markov model was used to align cichlid sequences to the 18 protein family alignment data bases currently available on the server. Q ss_pred: query secondary structure as predicted by PSIPRED; Q ss_conf: PSIPRED confidence values (0–9); Q query_name: query sequence; Q Consensus: query alignment consensus sequence; −quality of colum-column match (very bad = ; bad −; neutral .; good +; very good |; T Consensus: template alignment consensus sequence; T templ_name: template sequence; T ss_dssp: template secondary structure as determined by DSSP; T ss_pred: template secondary structure as predicted by PSIPRED; T ss_conf: PSIPRED confidence values (0–9); H: alpha-helix; E: extended beta sheet; C: coil; S: bend; B : beta brigded residue; T : turn; G : 3_10 helix residue.
(0.16 MB TXT)

Figure S4 Neighbor-Joining Phylogeny of MHC Class II B Functional Alleles. Neighbor-joining phylogeny of the 130 distinct alleles at 19 positively selected putative ABS amino acid sequences based on p-distances. Node bootstrap support (from 500 replicates) of more than 50% are indicated. Labeled clades were those used in intron 1 d and exon 2 dS comparisons.
(0.05 MB EPS)

Figure S5 Exon 2 and Intron 1 Allele Frequency Distributions in Pseudotropheus fainzilberi and P. emmiltos. Histograms of allele frequencies showing similar distributions for exon 2 amino acid sequences and intron 1 sequences. Private and shared alleles between the two species are also plotted separately. All alleles with an absolute frequency≥2 were included.
(0.07 MB EPS)

Table S1 Likelihood ratio tests comparing selective regimes affecting MHC class II B exon 2 in Pseudotropheus fainzilberi and P. emmiltos. Likelihood values under each of the alternative hypotheses were obtained from fitting models where species specific parameters were free to vary independently. Selective regimes were not significantly different between the species indicating that functional constraints were similar and therefore that selective pressures at individual sites could be inferred from the combined datasets.
(0.03 MB DOC)

Table S2 Empirical Bayesian posterior probabilities of belonging to each of the four substitution rate categories defined by a bivariate REL analysis. The selective pressures associated with each categories were defined as follow: purifying selection: dN/dS = 0.432; (nearly) neutral evolution: dN/dS = 1.107; positive selection dN/dS = 2.404, dN/dS = 5.746. Probabilities are reported for each of the 19 codons under positive selection of MHC class II β exon 2 in Pseudotropheus fainzilberi and P. emmiltos. Codon position numbers are those of the mature protein. Probabilities higher than 5% are highlighted in bold.
(0.04 MB DOC)

Table S3 Mean d, dS, and dN within clades of a neighbor joining tree built using amino acid p-distances at 19 positively selected putative ABS codons of MHC class II β. dS and dN were computed using the modified Nei and Gojobori method with a transition transversion ration of 1.26 (estimated from the data). d at intron 1 was measured using the Jukes and Cantor distance. Standard errors (SE) were computed from 500 bootstrap replicates. All calculations were done in MEGA v.3.1. Significantly (*α* = 0.05) different mean d in intron 1 and mean dS in exon 2 are marked in bold.
(0.06 MB DOC)
