# Supplementary material for: MHC Adaptive Divergence between Closely Related and Sympatric African Cichlids
Source: PLoS One. 2007 Aug 15;2(8):e734. doi: 10.1371/journal.pone.0000734 (PMC1939875; doi:10.1371/journal.pone.0000734)
Supplement: Table S1 — Likelihood ratio tests comparing selective regimes affecting MHC class II B exon 2 in Pseudotropheus fainzilberi and P. emmiltos. Likelihood values under each of the alternative hypotheses were obtained from fitting models where species specific parameters were free to vary independently. Selective regimes were not significantly different between the species indicating that functional constraints were similar and therefore that selective pressures at individual sites could be inferred from the combined datasets. (0.03 MB DOC) [file pone.0000734.s006.doc]

| Null hypothesis | LR | df | P-value |
| --- | --- | --- | --- |
| Same rate distribution (same dN/dS rate categories) | 3.877 | 10 | 0.953 |
| Same strength of selection (dN/dS) | 0.043 | 1 | 0.979 |
| Same proportion of sites with dN/dS > 1 | 0.597 | 1 | 0.440 |
| Same selective regime (same dN/dS and proportion of sites under selection | 0.645 | 2 | 0.724 |
