# Supplementary material for: MHC Adaptive Divergence between Closely Related and Sympatric African Cichlids
Source: PLoS One. 2007 Aug 15;2(8):e734. doi: 10.1371/journal.pone.0000734 (PMC1939875; doi:10.1371/journal.pone.0000734)
Supplement: Table S2 — Empirical Bayesian posterior probabilities of belonging to each of the four substitution rate categories defined by a bivariate REL analysis. The selective pressures associated with each categories were defined as follow: purifying selection: dN/dS = 0.432; (nearly) neutral evolution: dN/dS = 1.107; positive selection dN/dS = 2.404, dN/dS = 5.746. Probabilities are reported for each of the 19 codons under positive selection of MHC class II β exon 2 in Pseudotropheus fainzilberi and P. emmiltos. Codon position numbers are those of the mature protein. Probabilities higher than 5% are highlighted in bold. (0.04 MB DOC) [file pone.0000734.s007.doc]

|  | Substitution rate category (dN/dS) | | |  |
| --- | --- | --- | --- | --- |
| Codon position | 0.432 | 1.107 | 2.404 | 5.746 |
| 9 | < 0.0001 | < 0.0001 | < 0.0001 | **1** |
| 11 | < 0.0001 | < 0.0001 | **1** | < 0.0001 |
| 13 | < 0.0001 | < 0.0001 | **1** | < 0.0001 |
| 16 | < 0.0001 | < 0.0001 | < 0.0001 | **0.999** |
| 27 | < 0.0001 | < 0.0001 | < 0.0001 | **0.999** |
| 29 | < 0.0001 | < 0.0001 | **1** | < 0.0001 |
| 31 | < 0.0001 | < 0.0001 | < 0.0001 | **0.999** |
| 39 | < 0.0001 | 0.001 | < 0.0001 | **0.999** |
| 61 | < 0.0001 | < 0.0001 | 0.070 | **0.930** |
| 65 | < 0.0001 | < 0.0001 | < 0.0001 | **0.999** |
| 68 | < 0.0001 | < 0.0001 | **0.985** | 0.015 |
| 71 | < 0.0001 | < 0.0001 | < 0.0001 | **1** |
| 72 | < 0.0001 | < 0.0001 | **0.063** | **0.937** |
| 75 | < 0.0001 | < 0.0001 | < 0.0001 | **0.999** |
| 78 | < 0.0001 | 0.0003 | < 0.0001 | **0.999** |
| 81 | < 0.0001 | < 0.0001 | < 0.0001 | **1** |
| 82 | < 0.0001 | 0.0004 | < 0.0001 | **0.999** |
| 86 | < 0.0001 | < 0.0001 | **1** | < 0.0001 |
| 87 | < 0.0001 | < 0.0001 | **1** | < 0.0001 |
