# Supplementary material for: MHC Adaptive Divergence between Closely Related and Sympatric African Cichlids
Source: PLoS One. 2007 Aug 15;2(8):e734. doi: 10.1371/journal.pone.0000734 (PMC1939875; doi:10.1371/journal.pone.0000734)
Supplement: Table S3 — Mean d, dS, and dN within clades of a neighbor joining tree built using amino acid p-distances at 19 positively selected putative ABS codons of MHC class II β. dS and dN were computed using the modified Nei and Gojobori method with a transition transversion ration of 1.26 (estimated from the data). d at intron 1 was measured using the Jukes and Cantor distance. Standard errors (SE) were computed from 500 bootstrap replicates. All calculations were done in MEGA v.3.1. Significantly (α = 0.05) different mean d in intron 1 and mean dS in exon 2 are marked in bold. (0.06 MB DOC) [file pone.0000734.s008.doc]

|  | *P. emmiltos* | |  |  |  |  |  |  | *P. fainzilberi* | |  |  | |  | |  | |  |
| --- | --- | --- | --- | --- | --- | --- | --- | --- | --- | --- | --- | --- | --- | --- | --- | --- | --- | --- |
|  | Intron 1 |  | Exon 2 | |  |  |  |  | Intron 1 |  | Exon 2 | |  | |  | |  | |
| Clade | *d* | SE | *d*S | | SE | *d*N | SE |  | *d* | SE | *d*S | | SE | | *d*N | | SE | |
| 1 | **0.010** | 0.004 | **0.031** | | 0.012 | 0.083 | 0.019 |  | **0.057** | 0.009 | **0.024** | | 0.008 | | 0.063 | | 0.014 | |
| 2 | 0.025 | 0.009 | 0.046 | | 0.022 | 0.032 | 0.013 |  | **0.018** | 0.007 | **0.071** | | 0.022 | | 0.089 | | 0.022 | |
| 3 | 0.026 | 0.007 | 0.042 | | 0.017 | 0.127 | 0.027 |  | 0.026 | 0.013 | 0.039 | | 0.014 | | 0.100 | | 0.024 | |
| 4 | 0.007 | 0.004 | 0.000 | | 0.000 | 0.003 | 0.003 |  | 0.019 | 0.007 | 0.027 | | 0.014 | | 0.050 | | 0.013 | |
| 5 | **0.340** | 0.064 | **0.088** | | 0.022 | 0.143 | 0.027 |  | **0.314** | 0.047 | **0.101** | | 0.023 | | 0.174 | | 0.030 | |
| 6 | **0.386** | 0.070 | **0.085** | | 0.019 | 0.134 | 0.021 |  | **0.357** | 0.061 | **0.043** | | 0.015 | | 0.080 | | 0.017 | |
| 7 | 0.025 | 0.011 | 0.042 | | 0.017 | 0.037 | 0.011 |  | **0.170** | 0.039 | **0.044** | | 0.018 | | 0.050 | | 0.014 | |
| 8 | **0.191** | 0.028 | **0.052** | | 0.016 | 0.136 | 0.022 |  | 0.015 | 0.011 | 0.033 | | 0.017 | | 0.080 | | 0.022 | |
| 9 | 0.018 | 0.007 | 0.025 | | 0.012 | 0.036 | 0.010 |  | 0.065 | 0.010 | 0.037 | | 0.013 | | 0.085 | | 0.020 | |
| 10 | 0.020 | 0.008 | 0.017 | | 0.114 | 0.072 | 0.019 |  | 0.016 | 0.007 | 0.015 | | 0.009 | | 0.050 | | 0.016 | |
| 11 | **0.007** | 0.003 | **0.025** | | 0.010 | 0.040 | 0.010 |  | **0.008** | 0.004 | **0.059** | | 0.018 | | 0.104 | | 0.023 | |
| 12 | 0.015 | 0.007 | 0.030 | | 0.012 | 0.090 | 0.017 |  | 0.017 | 0.007 | 0.033 | | 0.012 | | 0.098 | | 0.019 | |
| 13 | 0.003 | 0.453 | 0.027 | | 0.012 | 0.024 | 0.005 |  | 0.294 | 0.137 | 0.028 | | 0.013 | | 0.067 | | 0.014 | |
| 14 | **0.149** | 0.019 | **0.026** | | 0.008 | 0.037 | 0.006 |  | **0.049** | 0.007 | **0.015** | | 0.005 | | 0.020 | | 0.005 | |
